# Evaluation of Chilled Dog Semen Extended With Sperm Activator

**DOI:** 10.3389/fvets.2021.764750

**Published:** 2022-02-11

**Authors:** Marcelo Martínez-Barbitta, Claudio Rivera Salinas

**Affiliations:** ^1^Postgraduate Program, Faculty of Veterinary Medicine, University of the Republic, Montevideo, Uruguay; ^2^Doctorate Program, Faculty of Veterinary Medicine, University of Perugia, Perugia, Italy; ^3^Reproductive Veterinary Service_Uruguay®, Nueva Helvecia, Uruguay; ^4^Clone® Chile – Reproductive Assistence Clinic, Santiago de Chile, Chile

**Keywords:** assisted reproductive biotechnologies (ART), sperm cryopreservation, chilled semen, semen activator extender (SA), andrology, sperm evaluation, longevity

## Abstract

Within modern biotechnology, different tools and methodologies have been developed to maximize canine semen conservation protocol to optimize reproductive results. In the last decades, the survival of chilled semen has been prolonged from 2 to 3 days with the first basic diluents, to 10–14 days with the modern extenders. However, their main limitation is that sperm quality decreases during cold storage. Sperm activators (SA) have been produced to provide the molecules necessary to maximize the sperm survival and quality with the aim to enhance fertility and prolificacy. In this study, the effect of commercial extender SA (Theriosolution® Canine AI extender -Chile-) was recorded by daily evaluation of chilled semen for 14 days. In this experiment, sperm-rich ejaculate fraction was collected from six adult healthy Neapolitan Mastiff dogs. The semen evaluation started immediately after collection (d0), and after that a next generation extender was added (d0) for every 24 h from d1 (with and without SA) to d14, to determine spermatozoa progressive motility, velocity of forward progression (VFP), morphology, and integrity of the spermatic membrane. The initial sperm concentration of extended semen was 417.3 ± 170.4 x 10^6^/mL (mean ± SEM) with 85.89 ± 4.76% of MNS (morphologically normal sperm), 84.47 ± 5.22 % live sperm, and pH of 6.2 ± 2.8. The initial VFP was 3.83 ± 0.48, but after 1 min with SA, it rises to 4.45 ± 0.45 (*P* < 0.001). The sperm progressive motility parameter increases significantly (*P* < 0.05) in experimental trial, respect to control, starting to d2 at finish (except for d7). The VFP analysis significantly increases in experimental trial (*P* < 0.05) during most days of the study with the exclusion of d3 and d14. To evaluate the seminal characteristics over time, the experiment was divided into T1 (d0–d5), T2 (d6–d10), and T3 (d11–d14) (*P* < 0.001) in evaluation of morphology and membrane functionality. The MNS reached 70% at d10 and finally 65% at d14, being considered normal and possibly fertile. With Host-s, 65% of MNS were also achieved at d14. The presence of glucose and fructose in the diluents used for refrigeration can exert very important effects given the fact that metabolic routes have been found in both sugars, providing both different and complementing effects. It can be concluded that the use of SA prior to artificial insemination improves the quality of chilled semen significantly, although it does not reverse the effects of deterioration due to cellular metabolism over time.

## Introduction

Despite the documentation of artificial insemination in dogs, for the first time, by Lazzaro Spallanzani in 1779, only in the last decades has its use in companion animals reproduction been more widely performed. Canine breeding increasingly requires the use of this technique using fresh, chilled, or frozen semen. The use of fresh, chilled, and frozen semen is indicated for many reasons in breeding management (1, 2).

Storage of chilled semen at 4–8°C induces a transition in the sperm plasma membrane from the cooled crystalline to the gel phase. At body temperature, the metabolism of the sperm is maximum, while at room temperature (24–29°C) it decreases. For every 10°C decrease, cellular metabolism is reduced by 50%; at 5°C metabolic activity of sperm is only 10% of what it would be at body temperature (3).

Semen extender must be used during the storage process of semen to limit the damages caused by temperature decline and to provide energy, maintain pH and osmolarity, reduce oxidation, and preserve plasma, acrosomal and mitocondrial membrane integrity, etc. (4–8).

Extenders used for cold storage of genetic material must provide cells with nutrients as an energy source; a buffer against damage by changes in pH; electrolyte concentration to control physiological osmotic pressure; and protection against bacterial growth and thermal shock during refrigeration and/or freezing (4, 5, 8). By contrast, activators are agents added, used in both chilled and frozen semen (although also with fresh semen), to enhance sperm activity and mainly have the function of providing rapidly available sugars for sperm cells as well as providing a buffer against intense metabolic activity that would imply an associated decrease of spermatozoa longevity and intrinsic fertility (9). They can also integrate other substances with preventive or conservative activities of the product itself or beneficial ones associated with semen handling (e.g., antibiotics).

More recently, sperm activators (SA) supplementations are used in extenders' basic composition. SA are easily metabolized carbohydrates that provide the mitochondria of the spermatic neck with a fast energy substrate to maximize their metabolism at the time of insemination; this has been studied by several authors (10–13) and enhances sperm progressive motility in order to maximize fertility and prolificacy of semen.

Therefore, use of long-term refrigeration extenders for semen together with activators when using the germplasm would enhance the management of these biotechnologies by the reproductive male, allowing the female to be managed more effectively, maximizing reproductive results (12–15). Therefore, the objective of this experiment was to evaluate the sperm progressive motility and survival of the spermatozoa under a dilution protocol with refrigeration for 14 days, corroborating the effect of SA during the whole process.

## Materials and Methods

### Animals and Location

Six Napolitan Mastiff male dogs with an average weight and age of 88 ± 12 kg (MED ± SEM) and 30.5 ± 3.5 months, respectively were used. The dogs were confirmed healthy based on history of proven fertility (litters in the last year), clinical examination including full andrological evaluation, and ultrasound examination of the prostate and testis. Dogs were fed twice daily (at 8:00 AM and 10:00 PM) using commercial dry food and selected chicken prey. Fresh water was available ad libitum.

The work was carried out in the month of December, at the facilities of the laboratory and semen bank of Clone® Chile (Santiago de Chile).

This experiment was approved by the bioethics and animal experimentation standards of the participating countries and has been evaluated by the corresponding committees.

### Semen Collection and Evaluation

Semen was collected by gloved hand techniques described previously (16) in the same day (d0) arround 8:00 PM and promptly examined the characteristic of seminal fluids, under a laminar flow chamber (Biobase® BBS-H1500, Chile). In particular the volume of the sperm-rich ejaculate fraction was determined, the pH of the seminal and prostatic fraction was assessed by pHmetro (Hanna Instrument® HI 5521, Chile), sperm morphology and vitality were assessed by eosin-nigrosine staining (Minitüb®, Tiefenbach, Germany) smears counting at least 200 spermatozoa per slide, and total sperm concentration was obtained by photometer (SDM 1 Photometer, Minitüb®, Tiefenbach, Germany, Series 1260162485, calibrated for canines) in accordance with manufacturer recommendations.

Moreover in the sperm-rich ejaculate fraction, the evaluation was performed, with two aliquots of 30 μL on slides, covered with coverslips, all pre-tempered at 37°C with thermal stage (HTM-MiniTherm, Hamilton® Thorne Biosciences—Beverly, MA, USA), for visualization using a phase contrast microscope (Olympus® BH-2 - Japan) X 100, waiting 60 s for observation, to determine progressive motility (%) and velocity of forward progression according to the scale described by Howard et al. (17). The evaluation was repeated at 5, 10, and 15 min.

After the initial evaluation of seminal parameters and within the first minute of collection of the second 30 μL semen aliquot, from d0 to d14, 30 μL of commercial extender SA (Theriosolution® Canine AI extender -Chile-) were added, were homogenized, and waited 60 s to reevaluate progressive motility and velocity of forward progression, as described above. The evaluation was also repeated at 5, 10, and 15 min.

In both fresh and chilled semen, sperm with a functional membrane were considered, which reacted to hypo-osmotic stress by dilating the distal part of the spermatic tail or curling it. While those sperm without changes in the tail were considered functionally damaged, and the results were expressed as a percentage of sperm with a functional membrane (sperm with abnormalities in the tail, by Farelly, were excluded from the count).

The individual semen samples were taken to a concentration of 100 x 10^6^ sperm per milliliter and were refrigerated at 4°C (Bozzo® SD-350 Refrigerator, Chile, with a maximum and minimum thermometer inside) and were evaluated in relation to their velocity of forward progression, progressive motility, morphology, and functionality every 24 h for 14 days (Commercial Diluent TherioSolutions® Canine Chilling, Chile). The alcohol in the outer tube avoided cold shock during the cooling process and temperature variations during the study period (18).

Every 24 h from extender addition, the structural and vital characteristics of the semen samples were evaluated after tempering for 60 s by the vital morphology stains of Farelly (Minitüb®, Tiefenbach, Germany), using the method described by Oettlé (19) X 1000 with immersion oil.

Sperm vitality due to membrane functionality was performed using the Hyposmolarity Test (Simplified Host –Host-s-), according to the method of Sánchez and Garrido (20).

### Characteristics of Diluents

The extenders used were commercial from TherioSolutions® laboratory. The composition declared by the manufacturer of TherioSolution® Canine AI extender is water, sodium citrate, TRIS, glucose, gentamicin, and proprietary factors. The composition published by the manufacturer of TherioSolutions® Canine Chilling is ultra-purified water, trisaminomethane, sodium citrate, glucose, gentamicin, and other undeclared components.

### Statistical Analysis

Normality and homogeneity of variances were verified using Shapiro-Wilk test. The results of thawed semen samples were expressed as mean ± standard error media by one-way analysis of variance (ANOVA). Duncan's *post-hoc* test was used to determine the differences between the groups using SPSS® 21.0 software (SPSS Inc., Chicago, IL, USA) (21). The difference between values was considered significant when the *P* < 0.05.

## Results

### Seminal Parameters

The volume of the second fraction of the ejaculate was 3.9 ± 1.6 mL with a pH of 6.2 ± 2.8. The prostatic fraction maintained the same value and dispersion in pH. The sperm concentration was 417.3 ± 170.4 million per mL. The initial evaluation of the sperm by eosin-nigrosin staining resulted in 85.89 ± 4.76 with no head staining, considering them to be alive.

### Analysis of Spermatozoa Velocity of Forward Progression (VFP)

**Figure 1:**
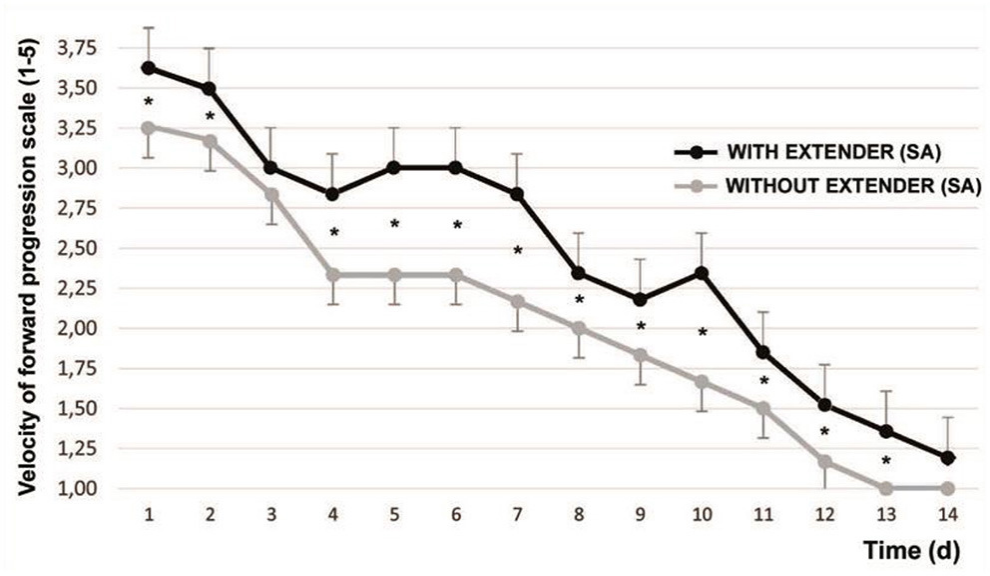
Variation of sperm velocity of forward progression [according to Howard et al. (17)] after cooling and timing. Mean ± SEM *P < 0.05. The graphed evaluations correspond to 1 min after adding SA.

The initial VPF (range 0–5) was 3.83 ± 0.48. After 1 min of SA, the VPF was 4.45 ± 0.45 (*P* < 0.001). Figure 1 shows the variation in the time of the refrigerated spermatozoa VFP after being adjusted at 37°C with and without the addition of SA.

From d1 to d13 they showed significant differences, with the exception of d3 (*P* < 0.065).

### Sperm Progressive Motility

**Figure 2:**
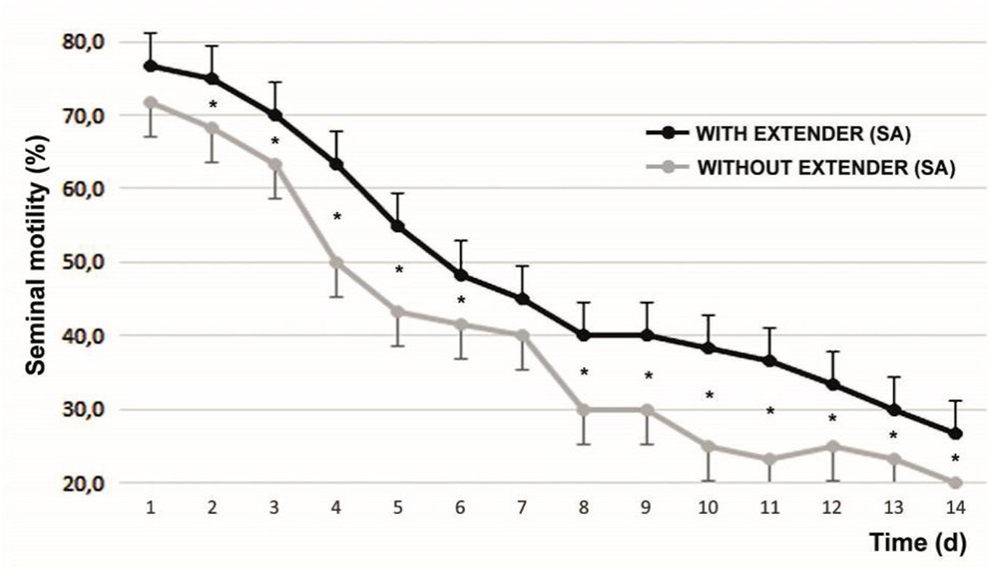
Progressive motility with and without the effect of seminal activator during the 14 d of the evaluation of refrigerated semen. Mean ± SEM *P < 0.05. The graphed evaluations correspond to 1 min after adding the SA.

The initial sperm progressive motility was 85.83 ± 5.23%. After 1 min of SA addition, progressive motility increased to 89.92 ± 5.18% (*P* < 0.05). Figure 2 shows the variability of sperm progressive motility with and without SA during the days of refrigeration of the semen. At all moments evaluated, with the exception of d1 and d7, significant differences were found between the samples.

**Figure 3:**
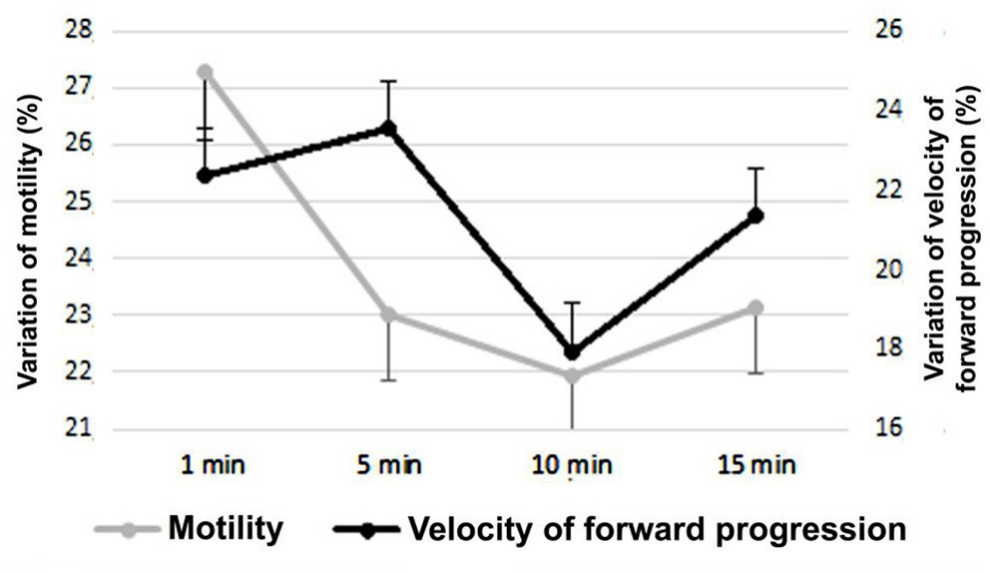
Velocity of forward progression and sperm progressive motility on day 14 after the placement of the sperm activator (Mean ± SEM). The variation was calculated as the difference between each value (progressive motility and velocity of forward progression) of each day with the course of the evaluation time (1, 5, 10, and 15 min).

In Figure 3 the variability of the average sperm velocity of forward progression and progressive motility of the entire period is observed, based on the differences according to the waiting of the evaluation, after the sample was tempered and refrigerated at 37°C. Since the functioning of the AS was unknown, it was evaluated during four different moments, in velocity of forward progression and motility evaluation.

According to the waiting period of the evaluation of the progressive motility and velocity of forward progression of the semen samples, after the placement of the SA, there were very important changes. At 1 min of placing the SA, the average velocity of forward progression during the 14 days was 2.43 ± 0.65, and at 15 min it increased to 3.23 ± 0.86 (*P* < 0.01), being 32.8% higher. For progressive motility, the variation was less but still substantially equal (*P* < 0.05), while the average evaluation of the period 1 min after adding the SA was 48.45 ± 12.9%; at 15 min it was 56.55 ± 15.11%, an increase of 16.7% at the end of the term.

### Spermatic Morphology

**Figure 4:**
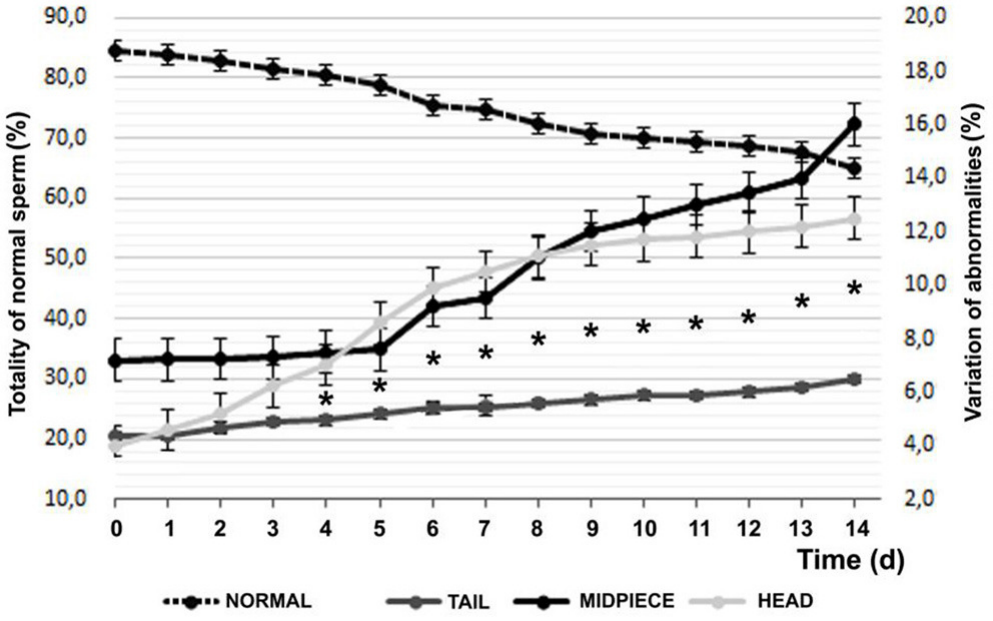
Temporal variation of sperm morphology by vital staining (Mean ± SEM). *Difference between of head or middle piece sperm defects and significant tail (*P* < 0.05).

Regarding morphology, 84.47 ± 5.22% of MNS (morphologically normal sperm), 3.96 ± 1.95 with head defects, and 7.20 ± 3.01 of defective intermediate piece were found with vital staining and finally 4.37 ± 0.61% of tail defects at the beginning of the experiment. Figure 4 observes the evolution of the defects and their distribution in the sperm during refrigeration.

After 14 days of decrease, with a practically linear decrease, a 19.47% lower value was found, with an average reduction of 1.5%/day, according to the Farelly staining analysis method. It should be noted that distinguishing between the affected area, tail changes increase by 49%, while in the intermediate part they increase by 122% and finally, problems in the sperm head grow by 216% (*P* < 0.001). Among the sperm anomalies found, at d14, 75.4% of the alterations in the sperm head corresponded to separate heads and acrosomes, while at d1 they constituted 20.2% (*P* < 0.05); in relation to the intermediate piece, the abnormal curvature and swelling was 32.6% of the total in d0 and the d14 (*P* = 0.055) rose to 80.2% and as for the tail, the abnormal curvature and the distal cytoplasmic gout went from 12.7% on d0 to 82.7% of total on d14 (*P* < 0.05).

### Membrane Functionality

**Figure 5:**
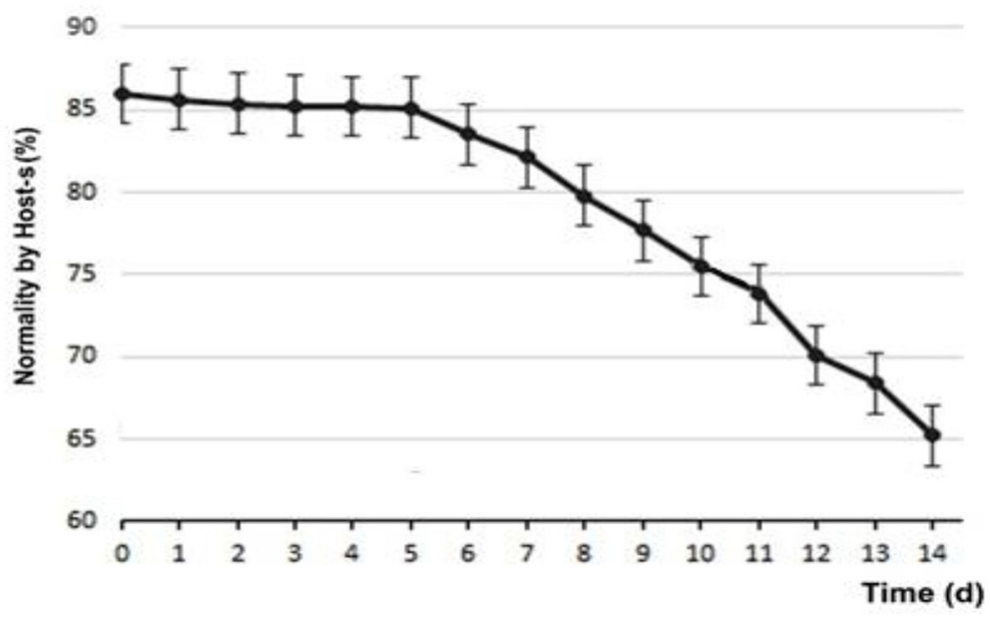
Variability of semen membrane functionality (Host-s) of refrigerated samples.

Regarding the functionality of the membrane, at d0 85.89 ± 4.76% of sperm were found to be normal, subtracting 14.11 ± 4.76% of those with permeability functionality defects. Figure 5 shows the variability of sperm membrane functionality.

To analyze the changes in membrane functionality and sperm morphology, the times were divided into three periods, according to the viability of the seminal extensors that are normally divided into short, medium, and long-term. Thus, T1 (short term) is used for the period between d0 and d5, T2 (medium term) for the period between d6 and d10, and finally T3 (long term) between d11 and d14. Table 1 shares the variations relative to the changes in sperm morphology and membrane functionality.

**Table 1:** Comparison between sperm morphology and membrane functionality test with distinction of storage times by refrigeration.

	**T1 (%)**	**CV (%)**	**T2 (%)**	**CV (%)**	**T3 (%)**	**CV (%)**
Normal Morphology^g^	81,97 ± 0,89^a^	2,67	72,62 ± 1,06^b^	3,28	67,62 ± 0,94^c^	2,78
Membrane functionality^h^	85,43 ± 0,13^d^	0,38	79,71 ± 1,45^e^	4,06	69,40 ± 1,81^f^	5,22

*T1 = d0–d5; T2 = d6–d10; T3 = d11–d14.*

*^g^Refers to Farelly's Vital Staining subjective assessment (total MNS).*

*^h^Corresponds to normal sperm according to Host-s.*

*Different letters in the same row p < 0.001*.

## Discussion

The volume, concentration, live and dead sperm (eosin/nigrosin), and pH of the second fraction of the ejaculate of all animals remained within normal values and therefore they were all included in the following analysis (22).

The objective of semen storage using chilling procedure is to preserve gametes at low temperatures, without reaching the freezing point, which induces deleterious intracellular changes that affect viability and fertilizing potency of sperm (9, 16). This technique of chilling preservation is a promising alternative to conventional semen cryopreservation and is easily adapted for clinical use. It is particularly useful for the shipment of semen where the costs of procedures and materials are high (23). This technique would also be of major importance if it extended the fertilizing potential of sperm for more than a few days (ideally between 10 and 14 d). Thus semen could be collected a few days after the detection of pro-estrus in a bitch and then processed, transported, and stored at the location of its intended use.

The commercial formulations of the laboratories reveal known components but also have components that are not disclosed in the formulas made by the manufacturers. TherioSolution® declares that it possesses glucose extender in SA extender and chilling products and fructose in the chilling formula. Most of the extensors reported for use in cryopreservation of domestic canine semen contain either glucose, lactose, or fructose (16, 18, 23–32). Furthermore, fructose has been used intensively in extenders for wild dogs such as foxes (24–27, 33). Sugars have long been included in semen diluents as exogenous energy substrates, as osmotic components, and as cryoprotective agents (34). Sperm can glycolyze glucose, fructose and mannose (35), and oxidize arabinose (36). However, the higher molecular weight of sugars such as lactose, sucrose, and raffinose have low permeability and are generally considered only as a cryoprotective agent (37) and not as an energy source. In dog semen, due to the absence of seminal vesicles, fructose is found in a very low concentration compared to that of other species (30, 38). However, canine sperm appear to be able to use fructose in a similar way to sperm from species with high fructose content in their accessory gland secretion. Still, it is important to notice that in 2001 Iguer-ouada and Verstegen found better results using glucose vs. fructose as the energy substrate, possibly indicating a preference of dog sperm to metabolize this molecule instead of fructose (23).

Ponglowhapan et al. (30) investigated the importance of the source and concentration of the type of energy source molecule in sperm metabolism during refrigeration in canines and found that lactose is more efficient than glucose in obtaining energy levels higher (ATP measurement enzymatically) in fresh semen. Furthermore, there are indications that fructose may possibly play a role as a sperm activator after ejaculation (39). Yildiz et al. (40) suggested that sugars allow the maintenance of osmotic pressure and perform a cryoprotective action. Based on this, it is expected that the inclusion of sugars in the extenders could serve as the preservation of the spermatic progressive motility, maintenance of the functionality, and integrity of the acrosome and the spermatic membrane (40, 41).

During our evaluation, in d14, either the partial results of the use of chilling extender using progressive motility and velocity of forward progression as indicators of vitality and Host-s as a test of plasma membrane functionality, showed very promising results. And at almost all times, the addition of the SA extender improved the quality of the evaluated semen, proving that this SA extender could be added at any time to stimulate quality and improve reproductive results. In the 14 days of evaluation, the MNS barely reached 70% at d10, reaching 65% at d14. These were able to be considered normal values that would not affect the fertility achieved with said semen (22). Analyzing the results of sperm morphology, divided into periods of time, some data to be highlighted emerge, taking all the altered sperm morphometries, between T1 and T2, it increases by 51.9%, while between T2 and T3, it increases by 18.3%, which means that, after d5, the variables normally standardized in most clinical evaluations practically stabilize, but there is a critical period from T1 into the future.

A high variability was found between the progressive motility and velocity of forward progression values achieved according to the evaluation time after the placement of the SA (Figure 3). From 1 to 15 min, the values always increased and had significant variations, which could be explained by the absorption of the seminal plasma membrane of the activator extender components over time, which takes some time and increases the metabolic activity of the sperm cell. It also establishes the importance of waiting time between the addition of the activator and the evaluation of the sample. This proves the necessity to be strict with the time frame of the evaluation and the methodology of the applied experimental design.

The use of Host-s, for hypo-osmotic stress or membrane functionality, has been validated in dogs for both fresh and refrigerated semen (20), in which the capacity of the membrane of the sperm cells to allow the flow of ions and of water into the cell is measured (42). Our results showed a functionality up to d5 of 85%, then it decreased almost linearly until d14 reaching 65%, decreasing ~2% per day. It was very interesting that after 2 weeks, two out of three sperm evaluated remained with plasma membrane functionality. Previous studies (43) had already shown that the addition of sugars significantly differentiated the response of sperm against hypo-osmotic tests in control samples in the absence of sugars. It reported that the progressive motility of sperm cells had a higher significant value with the use of refrigeration media with monosaccharides (glucose and fructose), compared to disaccharides (sucrose and trehalose), although some of these have shown possible membrane protective effects, compared to cryopreservation processes (14). This could be explained by their greater availability, either as an energetic medium for the spermatic neck mitochondria or as their cryopreservative function.

The results could be explained because during semen refrigeration, the main function of sugars is to provide the energy substrate required by sperm for the normal performance of their functions, glucose being one of the sugars best used by the sperm cell (41). Fernández-Novell et al. (44) observed in dog semen that glucose, but not fructose, can specifically activate the protein kinase AKT, involved in the regulation of several important cellular metabolic processes. This would imply that glucose would directly activate all AKT-regulated pathways of sperm. It can be assumed that, in the case of the canine, sugars can act not only as proper substrates of metabolism but also as direct modulators of spermatic function. The effects of these two sugars on the metabolism of freshly ejaculated sperm have been studied in dogs, and there is evidence that dog sperm metabolizes glucose and fructose using separate pathways (41). This results in differentiated management systems of energy as indicated by their different roles in glycogen metabolism (45), progressive motility patterns (39), hexose metabolism (41), and glycogen deposition (46).

Fructose, with respect to glucose, showed an increase in speed in the metabolic pathways and, therefore, in the formation of ATP. Fructose, by increasing the progressive motility-related consumption rate of ATP, results in a faster and more linear specific pattern than that observed with glucose (39), dedicating most of the energy consumption of the sperm to maintaining progressive motility. The effect of fructose on progressive motility would be related to a strong increase in the phosphorylation index of hexoses with respect to glucose. This increase, in addition to the consumption of ATP in the tyrosine phosphorylation, could lead to the establishment of the substrate that completes the cycle in which the energy that is immediately lost is generated. A drop in intracellular ATP levels would logically induce an immediate increase in ADP, which in turn would activate the glycolytic rhythm and increase ATP formation. When ATP returns to high levels, there would be a simultaneous drop in ADP levels, with a consequent decrease in glycolytic rhythm (47).

This feedback phenomenon would cause a high consumption rate and possible depletion of the fructose provided in the diluent, which indicates the importance of choosing the appropriate type and concentration of sugar, since small variations could cause large changes in the functional state and, therefore, the capacity of survival of sperm stored in refrigeration, hence the proposal analyzed by technicians and researchers to renew refrigeration diluents to maintain the metabolic activity of sperm and delay their death. In turn, the presence of both monosaccharides (glucose and fructose) is also highlighted in the extender for chilling used, where each monosaccharide would act differently on sperm cell metabolism, but only glucose in the SA extender.

Mammalian sperm require exogenous substrates for a variety of functions, for example, to preserve intracellular energy stores, cellular components, and most importantly, to support progressive motility (48). They can obtain energy through mitochondrial oxidative phosphorylation and glycolysis, by consuming glycolizable sugars, such as glucose, fructose, mannose, and maltose (49). Fructose is believed to be an important source of energy for ejaculated sperm (50), and along with glucose it is found in seminal plasma in many mammalian species.

The results of the present study clearly demonstrated that the main effect of glucose and fructose on cold semen extenders in canine are to provide inputs that intervene in sperm progressive motility and movement patterns. Progressive motility is an important indicator of the use of sugar by sperm since they provide the essential external energy source to maintain progressive motility. This is the practical criterion for evaluating semen quality on a commercial level, being widely associated with fertility (16).

There are not many studies that have published variations in seminal quality after prolonged periods (23, 30). Also, none have used SA in each period to assess the reaction of sperm to the addition of products that enhance their activity, finding more than important and significant reactions throughout the evaluation period, which could improve the viability of the cells and their mobility, which would ultimately affect the fertility and prolificacy obtained (49).

During d1, although there were apparently no modifications in the plasma membrane evaluated by Host-s, the morphological abnormalities increased significantly between 5 and 6%. Within these, especially those associated with problems in the sperm head (the abnormalities found were doubled from 4 to 8%, being highly significant), progressive motility decreased by 25% (highly significant) and velocity of forward progression decreased by one point. It is interesting to note that on average, the total variation of sperm abnormalities between days d1 and d14 were attributed to secondary or tertiary defects, associated with problems with the ability of sperm to remain anatomically viable during the 2 weeks of refrigerated storage, and are surely associated with the deterioration of membrane functionality after d5 (see Figure 5). These findings are consistent with those published by Ponglowhapan et al. (30), where these researchers found that in those first 5 days, the consumption of carbohydrates by the sperm cells was greater than a posteriori, which would indicate a deceleration of the metabolic rate of the sperm that could compromise the subsequent preservation and cell survival. Although possible changes in the capacitation and reactions of the sperm acrosome were not evaluated in the present work, several publications (26, 31, 51–53) reveal that the significance of these harmful effects in the sperm, which transcend cell death and lower fertility, are frequent in the freezing/thawing process, but not as pronounced in keeping the semen above the freezing point.

In this work, we found that the highest values of progressive motility and velocity of forward progression were obtained between 10 and 15 min after the activator was placed in the semen, when related to the evaluation at the moment of placing the SA, which would demonstrate the rapid availability of carbohydrates for cellular metabolism and the use of the latter by sperm.

No matter how much diluent is used to protect the sperm, heat shock will cause significant cell death. In our study, without the inclusion of the activator, there was a decrease of 32.91% in progressive motility and 41.45% in velocity of forward progression, initially, between d0 and d1, without the application of SA, data in the sense of previous research (23, 30).

## Conclusion

Refrigeration of semen for long periods, obtaining quality semen, allows the sending of samples over long distances, avoiding the mobilization of breeders with the associated stress, transportation costs, and potential health risks of mobility. The simplicity of the technique allows its mass use at a commercial level. The use of SA extender enhancers significantly improves the quality parameters at any time, after tempering the refrigerated sample, which would improve the fertility and prolificacy results of the obtained litters. These latter estimates require further studies to be verified.

The results obtained open the expectation to new working modalities with extenders for chilling that allow the use of breeding animals of high genetic value to be generalized, without the need to freeze the semen (with the dramatic changes that freezing/thawing cause in the sperm), with the complexities of transportation, and costs and handling that they require to obtain satisfactory results.

## Data Availability Statement

The raw data supporting the conclusions of this article will be made available by the authors, without undue reservation.

## Ethics Statement

Experimental procedures were approved by Animal Care Committee (Protocol Number 1420) and conducted in accordance with the guidelines of National Council of Animal Care. Written informed consent was obtained from the owners for the participation of their animals in this study.

## Author Contributions

MM-B: project administration, conceptualization, investigation, software, data curation, validation, and writing—reviewing and editing. CRS: financial support, methodology, and writing—original draft preparation. Both authors contributed to the article and approved the submitted version.

## Conflict of Interest

The authors declare that the research was conducted in the absence of any commercial or financial relationships that could be construed as a potential conflict of interest.

## Publisher's Note

All claims expressed in this article are solely those of the authors and do not necessarily represent those of their affiliated organizations, or those of the publisher, the editors and the reviewers. Any product that may be evaluated in this article, or claim that may be made by its manufacturer, is not guaranteed or endorsed by the publisher.
